# Factors associated with the appropriate use of asthma medications among adult asthmatic patients attending asthma clinic in a teaching hospital

**DOI:** 10.11604/pamj.2021.40.44.29137

**Published:** 2021-09-17

**Authors:** Victoria Sakyibea Aboagye, Kofi Adesi Kyei, Priscilla Awo Nortey, Doris Kitson-Mills, Joseph Daniels, Clement Korsah

**Affiliations:** 1 Department of Epidemiology and Disease Control, School of Public Health, College of Health Sciences, University of Ghana, Accra, Ghana,; 2 School of Biomedical and Allied Health Sciences, Department of Radiography, University of Ghana, Accra, Ghana,; 3 Korle-Bu Teaching Hospital, Accra, Ghana

**Keywords:** Asthma medication, treatment related factors, medication-related, patient-related

## Abstract

**Introduction:**

asthma tends to be more severe with worse symptoms in Africa due to late diagnosis and delayed initiation of treatment. To identify patient and treatment-related factors which influence the appropriate use of asthma medications.

**Methods:**

the study was institution based cross-sectional design. Patients were invited to provide information regarding the use of their asthma medications and factors potentially associated with appropriate use of these medications. A stepwise multivariate logistic regression analysis was used to evaluate the most important factor at a 0.05 level of significance.

**Results:**

respondents with better knowledge of their asthma medications were more likely to use them appropriately (OR 5.82 [CI 95% 2.25-15.04]) as were those with positive attitudes and beliefs towards asthma and asthma medications (OR 3.88 [CI 95% 1.44-10.44]).

**Conclusion:**

patients need to understand the importance of adhering to the prescribed regimen for their asthma medications even in the absence of overt symptoms in order to optimize clinical outcome.

## Introduction

Asthma is a chronic inflammatory airway disease characterized by intermittent, variable and reversible airway obstruction associated with airway hyper-responsiveness which results in recurrent episodes of wheezing, breathlessness, chest tightness and coughing [1]. Asthma is one of the major causes of chronic morbidity and economic burden of disease [2]. Asthma is a leading preventable cause of mortality and has been estimated to affect 300 million people worldwide, of which 50 million are in Africa [3]. It is estimated that more than 5% of the world's population suffer from asthma even as its prevalence continues to increase [2]. In Africa, the prevalence rate of asthma varies widely between different countries: Ethiopia 9%, Nigeria 13%, Mozambique 13.3%, Kenya 15.8% and South Africa 20.3% [4]. Although asthma has lower prevalence in sub-Saharan Africa, it is associated with more severe symptoms than in developed countries [5]. However, due to the projected increase in prevalence within the urban population in sub-Saharan Africa, it is estimated that there may be an increase of at least 35% in the number of people with asthma by 2025 [5]. Asthma is observed to be more severe in sub-Saharan Africa due to poor asthma care, late diagnosis and delayed initiation of treatment [5].

Empirical evidence indicates that acute exacerbations of asthma intensify when patients do not adhere to prescribed treatment regimen. This results in frequent hospitalizations and/or increased hospital visits to already overburden healthcare centers [6]. The frequency of asthma exacerbations can be reduced with effective therapeutic management.

In Ghana, the use of alternative medicine as well as factors such as health beliefs, attitudes and cultural practices of patients contribute to the incorrect use of prescribed medications [7] which leads to increased morbidity and mortality. There has been little documentation of factors that influence the appropriate use of asthma medications among adult asthmatic patients in Ghana. This research adds to existing literature by highlighting critical factors that lead to inappropriate use of asthma medications and also serves as a source of information for educating patients on how to use their medications appropriately. The aim of this study was to determine both patient and treatment-related factors associated with the appropriate use of asthma medications among adult asthmatic patients attending the asthma clinic of Korle-Bu Teaching Hospital (KBTH).

**Objectives:** the study sought to identify patient and treatment-related factors which influence the appropriate use of asthma medications. Specific objectives: 1) to identify the determinants of appropriate use of asthma medication; 2) to find the association between patient and medication related factors and appropriate use of asthma medication.

## Methods

**Study design and settings:** this research was an institution based cross-sectional study conducted over 6 weeks between December 2019 and January 2020 using a structured questionnaire. Adult patients were recruited into the study once every week, on Wednesday whilst attending the asthma clinic at Korle-Bu Teaching Hospital.

**Study participants and study size:** the study population included all adult patients who visited the asthma clinic during the period of the study. A total of one hundred and fifty (150) participants were recruited into the study using a convenient sampling method. The study included adults; 18 years and above, who have been diagnosed with asthma and have been prescribed a rescue (beta-agonist) and/or preventive (corticosteroid) inhaler. Only patients who had been seen at the clinic on at least two different occasions prior to the study were allowed to participate. Critically ill patients and those diagnosed with other chronic respiratory conditions such as Chronic Obstructive Pulmonary Disease (COPD) were excluded from the study.

**Variables:** socio-demographic variables included in this study were: age, sex and level of education. Independent variables which were included were: patients´ knowledge of medication, concerns about side-effects, frequency of dosing patients´ beliefs and attitudes towards disease and medication, duration of disease, patients´ knowledge of asthma and self-management of asthma, use of other daily medication, perceived benefits of medication, perceived need for medication and duration of use of medication. The dependent variable in this study was the appropriate use of asthma medication as prescribed by the healthcare provider.

**Data sources:** the tool used for the study was a modified questions generated from the works of Saldana *et al*. [8], Foster *et al*. [9] and Prabhakaran *et al*. [10]. A total of 42 items were used to evaluate patient-related factors such as attitudes, beliefs, perceived necessity for medication, availability of support relevant to treatment challenges, knowledge of symptoms and disease condition and inhaler technique (Annex 1, Annex 2). A combination of open and closed-ended questionnaires generated was utilized (Annex 2). The first section, included background information of respondents whereas the second section comprised patients´ asthmatic history and knowledge about their asthma medications. The third section assessed patients on medication-related factors and patient-related factors such as attitudes, beliefs, perceived necessity for medication, availability of support relevant to treatment challenges, knowledge of symptoms and disease condition and inhaler techniques. Self-reported use of asthma medication was compared with patients´ most recent medication as prescribed by the medical doctor to assess appropriate use of medication.

Patients were asked to name all of their asthma medications. For each of these medicines reported to be used, participants were asked when, how many times and how they took their medication. Using a 5-point Likert scale patients´ drug use was assessed. Patients were asked: if they took their medicines as prescribed, took their preventive medication prior to engaging in any strenuous activity or exercise, if they stopped taking their daily asthma medication when they felt better and if they forgot to take their preventive medication daily. Pretesting of the questionnaires was carried out on five adult asthmatic patients who attended the asthma clinic at the Tema General Hospital.

**Statistical methods:** the data collected was cleaned, anonymized, coded and imported into Statistical Package for the Social Sciences (SPSS) version 16 then exported to STATA 10 for analysis. The results were summarized by means of charts, graphs and frequency tables. Subsequently bivariate logistic analysis of association between appropriate use of asthma medications and the potential patient and medication-related factors considered in this study were performed. This was followed by multivariate logistic regression to evaluate important factors associated with appropriate use of asthma medication.

**Ethical consideration:** ethical clearance was obtained from the Ghana Health Service Ethical Review Board. Approval was also obtained from the Medicine and Therapeutic Department of the Korle-Bu Teaching Hospital. All recruited participants provided written informed consent.

## Results

Out of 141 eligible individuals, 97 provided informed consent and were subsequently interviewed, yielding a participation rate of 68.80%. Majority of the respondents interviewed were female 79 (81.45%). Patients´ age ranged between 18 and 87 with a mean age of 50.70 ± 16.96 years. About one-third of the participants were either traders or artisans and almost a quarter had retired. Also, about one-third of the participants had attained senior high school education whereas 7.2% had no formal education as shown in Table 1.

**Table 1:** socio-demographic characteristics of respondents, KBTH, 2019

Characteristics	N	%
**Age (years)**		
18 - 39	23	23.7
40 - 54	32	33.0
55+	42	43.3
**Mean age (years) ± SD = 50.70 ± 16.96**		
**Duration of asthma (years), mean ± SD = 22.24 ± 18.17**		
**Gender**		
Male	18	18.5
Female	79	81.5
**Highest level of education attained**		
No education	7	7.0
Primary level	13	13.0
Junior high school (JHS)	17	18.0
Senior high school**/**vocational	37	38.0
Tertiary level	23	24.0
**Occupation**		
Student	10	10.3
Professional	9	9.3
Artisan**/**traders	35	36.1
Unemployed	16	16.5
Clerical	7	7.2
Retired	20	20.6

All patients interviewed were on both short acting ß_2_- agonists (salbutamol) and a daily Inhaled Corticosteroids (ICS). The commonly used daily ICS included symbicort, serevent, pulmicort and seretide. In all, 12.37% of respondents had been recently prescribed oral prednisolone tablets. The mean duration of use of ICS was 3.1 ± 2.6 years. 25.8% of the respondents were on daily medication for other chronic conditions.

Overall, 37.1% of the participants were found to be using their medicines appropriately as indicated in Table 2. Again, in Table 2, 32% demonstrated good inhaler technique whereas 56.7% of the respondents self-reported appropriate use of their asthma medication. Only 7.2% were able to satisfy all three conditions. Of the 97 participants, 17.5% reported using their daily ICS once daily, 66.0% reported using it twice daily, 3.1% reported using it three times daily and the remaining 13.4% were not sure of how often they were to take their daily medication (Table 3). Of those using their asthma medication appropriately, 86.96% were females whilst 13.04% were males. Out of the 25 respondents who reported being on daily medications for other chronic conditions, only 7 were found to be appropriate users of their asthma medication.

**Table 2:** total number of patients fulfilling each criterion for appropriate use of asthma medication, KBTH, 2019

Criteria	Number (N)	Percentage (%)
Good inhaler technique*	31	32.0
Scoring a median cut of point of 16 or more (self-report)	55	56.7
Correct report and use of most recent prescription	36	37.1
Number satisfying all three criteria	7	7.2
Number satisfying any two of the criteria	36	37.1

* Getting steps 3 and 7 of both metered and dry powder inhaler techniques correctly

**Table 3:** a comparison of self-reported and required dosing regimen of ICS medication of participants, KBTH, 2019

		Frequency of dosing (daily)			
**Required medication regimen**		**Once**	**Twice**		
	N	4	93		
	%	4.1	95.9		
**Self-report medication regimen**		**Once**	**Twice**	**Three times**	**Not sure**
	N	17	64	3	13
	%	17.5	66.0	3.1	13.4

Six (6) patient-related factors were considered in this study. Majority (55.7%) of the respondents demonstrated poor knowledge of their asthma medications. Over half (56.7%) of the respondents had positive attitudes and beliefs towards asthma and their medications. A greater proportion (78.4%) of the participants did not report any experiences of side effects with use of asthma medication. A significant portion of the respondents agreed that medical support was available whenever they needed it (77.3%).

**Determinants of appropriate use of asthma medication:** none of the socio-demographic or background characteristics were found to be significantly associated with appropriate use of asthma medication. Bivariate analysis of age and appropriate use of asthma medication did not show any significant association (p=0.75). This observation was not different across the stratified age groups. Sex was also not significantly associated with appropriate use of asthma medication (p=0.44). The stratified educational levels were not found to be significantly associated with appropriate use of asthma medication. Appropriate use of asthma medication did not improve with number of years since diagnosis or number of years of asthma treatment.

**Association between patient and medication related factors and appropriate use of asthma medication:** of the 10 patient and medication related factors considered, 2 patient-related factors were retained in the final multi-variate logistic regression model. None of the medication-related factors were associated with appropriate use of asthma medication. Good knowledge of asthma medication was statistically significant when associated with appropriate use of asthma medication in both bivariate and multivariate analysis (p=0.001). Participants who were more knowledgeable of their medications (Table 4) were more likely to use their medication appropriately (OR 5.82 [95% CI 2.25-15.04]) as were those with positive beliefs and attitudes towards asthma and asthma medication (OR 3.880 [95% CI 1.44-10.44]).

**Table 4:** factors associated with appropriate use of asthma medication, KBTH, 2019

Factors	Unadjusted OR (95% CI)	P-value	Adjusted OR (95% CI)	P-value
Knowledge of asthma medication	5.43 (2.21 - 13.32)	<0.000	5.82 (2.52 - 15.04)	<0.001
Beliefs and attitudes toward asthma and medication	3.54 (1.43 - 8.76)	0.006	3.88 (1.44 - 10.44)	0.007

CI: confidence interval

## Discussion

The low proportion (37.1%) of sampled adult asthmatic patients found to use their asthma medications appropriately is consistent with other studies that found that the use of asthma medication is suboptimal and that, in chronic diseases adherence to daily medication regimen remains a challenge [11,12]. The principal finding in this study was the identification of two main patient-related factors associated with the appropriate use of asthma medications. The study also revealed that patients with positive attitudes and beliefs towards asthma and their medication were more likely to use their medication appropriately. This correlates with findings in similar studies which showed that suboptimal beliefs about asthma could interfere with the appropriate use of medication leading to poor clinical outcome in asthmatic patients [13].

Some of the participants admitted that they felt shy to take their medication in public stating that they were concerned about people stigmatizing them for not being normal healthy people. A majority of the patients interviewed had been visiting the clinic for years and therefore a higher proportion of the respondents were expected to use their medication appropriately. This was however not the case, suggesting that patients had underlying beliefs which had caused them to develop certain attitudes towards asthma which had in turn become an obstruction in optimizing use of medication. This may be partly due to lack of effective education on the part of health workers or patients´ own way of dealing with asthma. A majority of the participants were females which is typical of attendance at the clinic (hospital records, KBTH). This reflects the relatively higher prevalence of asthma among adult females compared to males [14]. No sociodemographic factor was found to be associated with appropriate use of asthma medications. Similar to previous comparable studies, age and gender were found to be poor predictors of either appropriate or inappropriate use of asthma medication [15-17]. Unlike some previous studies which reported that cost plays a key role in predicting improved use of medication [16], this study did not identify any such association between cost and use of medication. Most participants in this study indicated that access to asthma medications was generally not a problem because these medications were covered by the National Health Insurance Scheme (NHIS).

This study showed that years of being with asthma and years of use of asthma medication did not translate into appropriate use of asthma medication and this support other studies by De Smet *et al*. [18] and Santos *et al*. [19]. Naturally, it would be expected that patients with higher educational qualification should have better knowledge of their condition and medication which should translate into improved use of their medications. However, this study found no association between the level of formal education and appropriate use of asthma medications. Surprisingly, another study demonstrated that lower educational status was associated with improved use of asthma medication [20].

About 56.7% of participants self-reported improved use of their asthma medication. A breakdown of the criteria used in assessing appropriate users of asthma medication revealed that just about one third demonstrated good inhaler technique. A similar proportion gave correct details of their most recent prescribed medication as shown in them Table 3. This suggests that though a majority of the respondents self-reported appropriate use of their medication, a sizable number did not fully comprehend what had been prescribed them, and were therefore not likely to have used their medications appropriately. All 95.9% of the respondents were expected to use their ICS twice daily. However, only 66.0% confirmed this dosing frequency. Some participants were not even sure of how often they were to take their daily ICS, indicative of the fact that they did not use them as prescribed.

Previous studies have demonstrated that side effects experienced by patients deterred them from using their medication appropriately [9]. Majority of the patients did not report experiences of side effects (78.4%) and therefore it was not associated with appropriate use of asthma medication. The few who reported adverse effects were not sure which medication was responsible for what they had experienced. This was suggestive that not much education or emphasis had gone into this aspect of pharmaceutical care which is the responsibility of pharmacists.

This study did not capture other factors which could also have been associated with appropriate use of medications. However, several studies have demonstrated that patient and medication-related factors were mostly the key factors identified as predictors of appropriate use of medications [19,21]. Factors such as healthcare team and system-related factors are usually complex to study due to the challenges associated with its measurement [13]. The two patient-related factors found to be associated with appropriate use of asthma medication indicate that patients have a responsibility in ensuring that they optimize the treatment given to them. This can however not be achieved without expert advice, counselling and education from healthcare providers.

**Limitations of the study:** this study was carried out at Korle-Bu Teaching Hospital and therefore the results may not be generalizable to other clinical settings where clinical practice and patient care may be different. The study was carried out over six weeks and other patients who may have different characteristics could have been missed however, considering the fact that each patient is seen at most four times in a year, it is likely that not too many of such individuals were missed. The cross-sectional design of this study did not permit causal inferences to be made and therefore precedence of the associated factors must be assumed. Notwithstanding these limitations the information gathered from this study is valuable for understanding likely factors that influence appropriate use of asthma medication among adult asthmatic patients in general.

## Conclusion

The proportion of adult asthmatic patients attending the asthma clinic at Korle-Bu Teaching Hospital who appropriately use their asthma medication was 37.1%. Patients with sound knowledge of their asthma medication were likely to have had better understanding of their medication and therefore more likely to use their asthma medications appropriately. Respondents with positive attitudes and beliefs towards asthma and their medications were more likely to use them appropriately. The findings in this study indicate the need for innovative but cost-effective interventions with focus on educating patients on their medications and helping them to understand the need to use their asthma medications appropriately as prescribed. Pharmacists at the health facility must educate patients and offer comprehensive pharmaceutical care in order to reshape the health beliefs of patients and maximize medication use. Future studies may focus on replicating this study in other clinical settings to confirm these findings and to explore other factors not considered in this study that may assist in addressing the inappropriate use of asthma medications among adults.

### What is known about this topic

- Though the prevalence of asthma in sub-Saharan Africa is lower than in developed countries, the disease is known to be severe in Africa due to poor asthma care, late diagnosis and initiation of treatment;
- Research indicate that medication is an integral aspect of asthma management;
- Empirical evidence indicates that acute exacerbations of asthma intensify when patients do not adhere to asthma treatment regimens.

### What this study adds

- No sociodemographic factor was found to be associated with appropriate use of asthma medications;
- Patients with positive attitudes and beliefs towards asthma and their medication were more likely to use their medication appropriately;
- Years of being with asthma and years of use of asthma medication did not translate into appropriate use of asthma medication.

## Annexes

**Annex 1**: check list used in assessing inhaler technique (PDF-62Kb)

**Annex 2**: sample questionnaire: factors associated with appropriate use of asthma medication among adult asthmatic patients attending the asthma clinic of Korle-Bu Teaching Hospital (PDF-133Kb)
